# Nicotinate-curcumin improves NASH by inhibiting the AKR1B10/ACCα-mediated triglyceride synthesis

**DOI:** 10.1186/s12944-024-02162-5

**Published:** 2024-06-27

**Authors:** Xiu-lian Lin, Ya-ling Zeng, Jie Ning, Zhe Cao, Lan-lan Bu, Wen-Jing Liao, Zhi-min Zhang, Tan-jun Zhao, Rong-geng Fu, Xue-Feng Yang, Yong-zhen Gong, Li-Mei Lin, De-liang Cao, Cai-ping Zhang, Duan-fang Liao, Ya-Mei Li, Jian-Guo Zeng

**Affiliations:** 1 Key Laboratory for Quality Evaluation of Bulk Herbs of Hunan Province, Hunan University of Chinese Medicine, Changsha, 410208 Hunan China; 2 Hunan Laituofu Biotechnology Co., Ltd, Jinzhou New District, Ningxiang, 410604 Hunan China; 3 Hunan Provincial Clinical Research Center for Metabolic Associated Fatty Liver Disease, Hengyang, 421002 Hunan China; 4 Hunan Key Laboratory of Traditional Chinese Veterinary Medicine, Hunan Agricultural University, Changsha, 410128 Hunan China; 5 Department of Biochemistry & Molecular Biology, Hengyang Medical School, University of South China, Hengyang, 421001 Hunan China; 6 Present Address: Department of Endocrinology, Shenzhen Longhua District Central Hospital, Guangdong Medical University Affiliated Longhua Central Hospital, Shenzhen, 518110 Guangdong China

**Keywords:** Nicotinate-Curcumin, NASH, AKR1B10, Triglyceride

## Abstract

**Background:**

Nonalcoholic steatohepatitis (NASH) is a prevalent chronic liver condition. However, the potential therapeutic benefits and underlying mechanism of nicotinate-curcumin (NC) in the treatment of NASH remain uncertain.

**Methods:**

A rat model of NASH induced by a high-fat and high-fructose diet was treated with nicotinate-curcumin (NC, 20, 40 mg·kg^− 1^), curcumin (Cur, 40 mg·kg^− 1^) and metformin (Met, 50 mg·kg^− 1^) for a duration of 4 weeks. The interaction between NASH, Cur and Aldo-Keto reductase family 1 member B10 (AKR1B10) was filter and analyzed using network pharmacology. The interaction of Cur, NC and AKR1B10 was analyzed using molecular docking techniques, and the binding energy of Cur and NC with AKR1B10 was compared. HepG2 cells were induced by Ox-LDL (25 µg·ml^− 1^, 24 h) in high glucose medium. NC (20µM, 40µM), Cur (40µM) Met (150µM) and epalrestat (Epa, 75µM) were administered individually. The activities of ALT, AST, ALP and the levels of LDL, HDL, TG, TC and FFA in serum were quantified using a chemiluminescence assay. Based on the changes in the above indicators, score according to NAS standards. The activities of Acetyl-CoA and Malonyl-CoA were measured using an ELISA assay. And the expression and cellular localization of AKR1B10 and Acetyl-CoA carboxylase (ACCα) in HepG2 cells were detected by Western blotting and immunofluorescence.

**Results:**

The results of the animal experiments demonstrated that NASH rat model induced by a high-fat and high-fructose diet exhibited pronounced dysfunction in liver function and lipid metabolism. Additionally, there was a significant increase in serum levels of FFA and TG, as well as elevated expression of AKR1B10 and ACCα, and heightened activity of Acetyl-CoA and Malonyl-CoA in liver tissue. The administration of NC showed to enhance liver function in rats with NASH, leading to reductions in ALT, AST and ALP levels, and decrease in blood lipid and significant inhibition of FFA and TG synthesis in the liver. Network pharmacological analysis identified AKR1B10 and ACCα as potential targets for NASH treatment. Molecular docking studies revealed that both Cur and NC are capable of binding to AKR1B10, with NC exhibiting a stronger binding energy to AKR1B10. Western blot analysis demonstrated an upregulation in the expression of AKR1B10 and ACCα in the liver tissue of NASH rats, accompanied by elevated Acetyl-CoA and Malonyl-CoA activity, and increased levels of FFA and TG. The results of the HepG2 cell experiments induced by Ox-LDL suggest that NC significantly inhibited the expression and co-localization of AKR1B10 and ACCα, while also reduced levels of TC and LDL-C and increased level of HDL-C. These effects are accompanied by a decrease in the activities of ACCα and Malonyl-CoA, and levels of FFA and TG. Furthermore, the impact of NC appears to be more pronounced compared to Cur.

**Conclusion:**

NC could effectively treat NASH and improve liver function and lipid metabolism disorder. The mechanism of NC is related to the inhibition of AKR1B10/ACCα pathway and FFA/TG synthesis of liver.

**Supplementary Information:**

The online version contains supplementary material available at 10.1186/s12944-024-02162-5.

## Introduction

Nonalcoholic steatohepatitis (NASH) represents the advanced phase of nonalcoholic fatty liver disease (NAFLD) and is considered a more severe manifestation of the condition [1, 2]. The hepatic steatosis observed in NAFLD is believed to result from the excessive accumulation of triglycerides (TG) within hepatocytes, stemming from impaired secretion or metabolic dysregulation [3]. These abnormalities encompass disruptions in glucose homeostasis, and disturbances in fatty acid (FA) and TG signaling pathways, encompassing synthesis, transport, and oxidation processes [4]. Disturbance in the equilibrium of fat uptake, synthesis, and clearance within the liver can result in the progressive buildup of FFA, TG, and pro-inflammatory agents, culminating in hepatic steatosis [5]. This condition may advance from benign steatosis to NASH, marked by hepatic lipid accumulation, inflammation, and fibrosis [6]. While reversible and relatively benign, simple steatosis may progress to irreversible liver cirrhosis and malignancy in advanced stages of NASH, posing a significant health risk. The upregulation of FFA and TG synthesis represents the principal pathological and biochemical mechanism underlying NASH, serving as a pivotal etiological factor. As NAFLD progresses to NASH, abnormalities in certain indicators, such as TM6SF2 [7, 8] and AKR1B10 [9], will be crucial focal points for monitoring free fatty acid and lipid synthesis. Specifically, the process of synthesizing FFA and TG involves the conversion of Acetyl-CoA to Malonyl-CoA through the action of ACCα carboxylation. Malonyl-CoA can then react with FFA to produce long-chain fatty acids with the assistance of various enzymes. These long-chain fatty acids can subsequently combine with glycerol to form TG [10]. AKR1B10 is primarily expressed in gastric and intestinal epithelial cells under physiological conditions, exhibiting minimal to no expression in other tissues [11]. In the context of cancer, NAFLD and skin lesions, AKR1B10 exhibits high expression in associated tissues. Research indicates that AKR1B10 plays a role in facilitating the ubiquitin-dependent degradation of ACCα, thereby inhibiting its ubiquitination and proteolysis, ultimately promoting fatty acid and lipid synthesis [12, 13]. Notably, studies have demonstrated abnormal up-regulation of AKR1B10 during the progression from NAFLD to nonalcoholic steatohepatitis (NASH) [14, 15].

Currently, the primary pharmacological interventions for NAFLD/NASH consist of pioglitazone, metformin, and vitamin E; however, their efficacy is limited due to a low therapeutic index and notable adverse effects. For instance, pioglitazone has been associated with heart failure, osteoporosis, and weight gain [16]. The absence of a definitive treatment for NASH underscores the pressing need for the development of novel pharmaceutical agents to address and manage NAFLD/NASH [17]. Herbal remedies and phytochemicals have emerged as adjunctive and alternative modalities for liver disorders, including NAFLD, with numerous preclinical investigations and clinical trials currently underway [18]. Nevertheless, the likelihood of achieving success remains limited. Consequently, there is a need for ongoing investigation into pharmaceuticals that exhibit strong efficacy and well-defined targets. Prior research has demonstrated the effectiveness of traditional Chinese medicine, including Huanglian [19], Huangqi [20], Yuzhu [5], and Gegen Qinlian decoction [21], in treating NAFLD/NASH. Curcumin (Cur), a hydrophobic polyphenol derived from turmeric, has been found to markedly decrease TG levels in rats with fructose-induced NAFLD and effectively ameliorate fatty liver and fibrosis [22, 23]. Notably, Cur has the ability to modulate the AKR1B10 protein.

A recent development in the field of pharmacology involves the creation of a series of curcumin derivatives with potential applications in the regulation of lipid metabolism disorders [24–26]. Among these derivatives is nicotinate-curcumin (NC), a novel compound synthesized by the author’s team that combines the basic structure of curcumin with the addition of a niacin group, showing preferable properties of lipid-regulation and anti-inflammatory (Chinese patent CN101805285A) [27, 28]. Our previous research demonstrated that NC have notable effects on lipid metabolism and anti-atherosclerosis [25, 29, 30], surpassing the effects of curcumin [28, 30]. Therefore, we propose that NC may impede the progression from NAFLD to NASH by suppressing the synthesis of FFA and TG. In this study, we investigated the effects of NC on NASH and its potential mechanism involving the inhibition of AKR1B10/ACCα in regulating TG synthesis by utilizing a rat NASH model induced by a high-fat and high-fructose diet, as well as a hepatocyte model induced by Ox-LDL in high glucose medium.

## Materials and methods

### Feed and reagent

The rat model induction feed, procured from Nutritious Animal Feed High-tech Co., Ltd. (Nantong, Jiangsu) and stored at 4℃. The main components of the basic feed include wheat flour, corn meal, secondary meal, soybean meal, corn gluten meal, vegetable oil, beer yeast, salt, lysine, methionine, minerals and vitamins. Notably, the composition of this feed closely resembles that of Harlan 2018. The formula for high-fat and high-fructose induction feed is as follows: cholesterol 1%, lard 10%, sodium cholate 0.5%, basic feed 88.5%. 20% fructose was added to drinking water.

The blood lipid test kit was purchased from Zhejiang Eastern Europe Diagnostic Co., Ltd. (Wenzhou, Zhejiang). OCT embedding agent and paraformaldehyde were purchased from Solebar Biotechnology Co., Ltd. The kits of alanine aminotransferase (ALT), aspartate aminotransferase (AST) and alkaline phosphatase (ALP) were purchased from Nanjing Jiancheng Institute of Biological Engineering. The Acetyl-CoA activity test kit (BC0980) was purchased from Solebar Biotechnology Co., Ltd., and the Malonyl-CoA (QC6538) activity test kit was purchased from Shanghai Qincheng Biotechnology Co., Ltd. In addition, hematoxylin, eosin, xylene, ethanol and isopropanol are all prepared in the laboratory. AKR1B10 (AB96417) antibody was purchased from American Abcam company, ACC (3676) antibody was purchased from CST company (USA), and β-actin second antibody was purchased from Proteintech Group, Inc. (China). Epalrestat (B21492) was purchased from Shanghai Yuanye Bio-Technology Co., Ltd.

### Animal experiment and cell experiment

#### Establishment of NASH rat model induced by high fat - high fructose

Male SD rats, weighing approximately 200 g were purchased from the Hunan Experimental Animal Center and housed in a specific pathogen-free (SPF) animal laboratory, possessing animal production license number SCXK (Xiang) 20,160,001 and animal license number SYXK (Xiang) 2015-0016.

Sixty-six Sprague-Dawley rats were randomly allocated into a blank control group (Ctrl), a model group (HFD + Fru), a metformin control group (Met), a curcumin treatment group (Cur), a low-dose nicotinate-curcumin group (NC-L), and a high-dose nicotinate-curcumin group (NC-H), 11 rats per group.

**Fig. 1:**
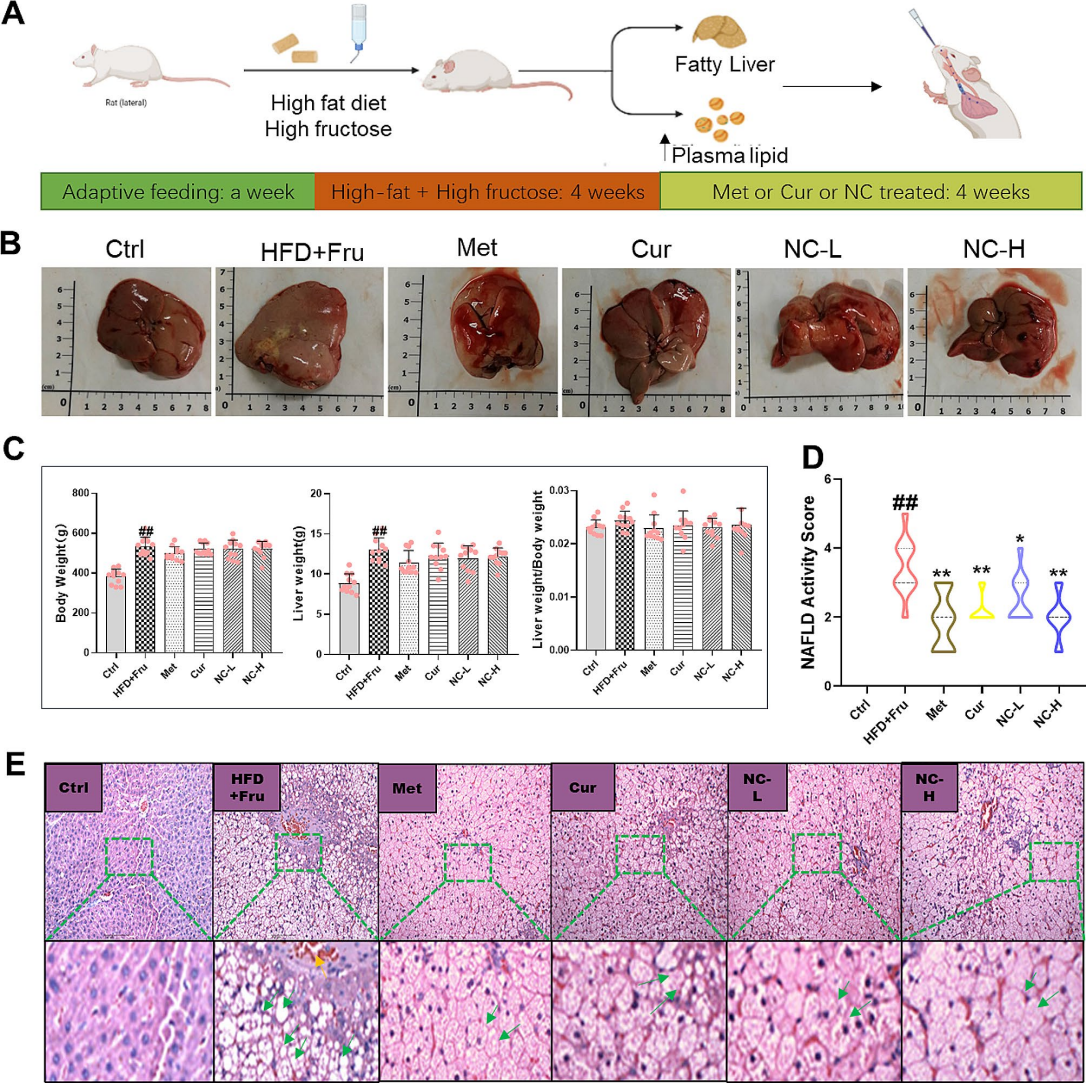
Effects of nicotinate-curcumin (NC) on hepatic index and pathological alterations in high-fat and high-fructose diet-induced NASH rats. A rat model of NASH induced by HFD + Fru was used to treat rats by intragastric administration. The dose of Met was 50 mg·kg^− 1^, the dose of Cur was 40 mg·kg^− 1^, and the dose of NC was 20 mg·kg^− 1^ and 40 mg·kg^− 1^. (**A**) Analytic diagram of experiment process; (**B**) Rat liver diagram; (**C**) Body weight, liver weight and liver weight/body weight ratio of rats; (**D**) NAFLD activity score (NAS). (**E**) HE staining of rat liver. The data is expressed in mean ± SD. ^#^*P* < 0.05 and ^##^*P* < 0.01 vs., control (Ctrl). ^*^P < 0.05 and ^**^P < 0.01 vs. High fat diet + Fructose (HFD + Fru)

Although NAFLD represents relatively simple steatosis in the early stage of fatty liver, NASH represents a more severe and irreversible progression of the disease that can lead to cirrhosis and hepatocellular carcinoma [31]. The animal model fed with high-fat and high-fructose diet showed typical NAFLD steatosis after 4 weeks, followed by typical NASH features including globular hepatocyte degeneration, diffuse lobular inflammation, and fibrosis after 8 weeks [32]. Thus, nicotinate-curcumin (NC-L, 20 mg·kg^− 1^; NC-H, 40 mg·kg^− 1^) was administered in the fifth week following a four-week period of high fat and high fructose diet, while curcumin (Cur, 40 mg·kg^− 1^) and metformin (Met, 50 mg·kg^− 1^) were employed as comparative treatments. Feeding regimens were sustained for an additional four weeks (Fig. 1A), with the normal control group receiving a standard compound feed.

#### Cell culture

HepG2 cells obtained from Wuhan Shangen Biotechnology Co., Ltd. were cultured in MEM medium supplemented with 10% newborn calf serum and 1% penicillin/streptomycin at 37℃ in a 5% CO_2_ humidified incubator. The cells were exposed to oxidized low density lipoprotein (Ox-LDL) at a concentration of 25 µg·ml^− 1^ for 24 h, followed by co-induction in high glucose medium (DMEM) to establish a cell high fat model consistent with animal experiments. Subsequently, the cells were treated with nicotinate-curcumin (NC, 20µM, 40µM), curcumin (Cur, 40µM), epalrestat (Epa, 75µM) and metformin (Met, 150 µM) for a duration of 24 h.

### Experimental method

#### Assessment of serum samples and liver tissues for the identification of associated biological markers

Evaluate the effectiveness of prevention and treatment at the end of 8 weeks of high-fat and high fructose feeding. Prior to sampling and weighing, the animals underwent a 12-hour fast. Subsequently, the abdominal cavity of the rats was opened to examine the overall morphology of the liver, including its size and hue. Blood samples were obtained from the abdominal aorta and centrifuged at 4℃ and 3000 rpm for 10 min.

Serum and EDTA anticoagulant plasma were collected and stored at -80℃ for the detection of serum ALT, AST, ALP, TC, TG, HDL, LDL, and FFA levels. Liver tissue samples from each rat were homogenized using a high-speed, low-temperature homogenizer and centrifuged at 3000 rpm for 10 min. Subsequently, tissue supernatants were utilized for the quantification of Acetyl-CoA and Malonyl-CoA levels following the manufacturer’s instructions for the respective kits. The remaining portion of the liver tissue was preserved in 4% paraformaldehyde, dehydrated, embedded in paraffin, sectioned, stained with standard hematoxylin and eosin, and sealed. Examination under a light microscope revealed the presence of hepatocyte degeneration index (including edema, steatosis, and eosinophil degeneration), necrosis, hepatic sinusoidal dilatation, blood stasis syndrome, fibrous tissue hyperplasia, and inflammatory cell infiltration.

#### Assessment of hepatic function and serum lipid profiles

The levels of ALT, AST, ALP, TG, TC, LDL-C, HDL-C, and FFA were quantified following the prescribed sample preparation procedures and guidelines provided by the manufacturers. The assays for Acetyl-CoA and Malonyl-CoA activity were conducted in adherence to the instructions outlined by the respective kit manufacturers.

#### Western blotting analysis

100 mg of liver tissue samples were collected from the same region of the liver and subsequently washed with phosphate-buffered saline (PBS) at 4℃. The tissues were then homogenized using RIPA buffer supplemented with an enzyme inhibitor (RIPA 1:100) and subjected to three rounds of grinding at 4℃ for 45 s each, with a 20-second interval between each round. Following homogenization, the cells were washed three times with PBS at 4℃ and then lysed at low temperature using RIPA buffer containing a protease inhibitor for 30 min. The lysed cells were transferred to an EP tube and subjected to 2–3 rounds of sonication, after which the supernatant was centrifuged at 16,000 g for 15 min. Total protein was quantified using a BCA kit. Briefly, each sample was treated with bromophenol blue buffer and denatured at 100 °C for 10 min. The target protein was then separated using SDS-PAGE gel in Western blotting analysis, transferred to a PVDF membrane, and blocked with 5% milk or 5% bovine serum albumin in TBST for 1 h. Subsequently, the primary antibody specific to AKR1B10 (1:1000) or ACCα (1:1000) was added and incubated overnight at 4 °C. The following day, the secondary antibody corresponding to the host species of the primary antibody was incubated at 37 °C for 1 h.

#### Predicting the migration and interaction of cur with key targets in NASH through network pharmacology

The active components of curcumin were obtained from the TCMSP and TCMID databases, while the two-dimensional and three-dimensional structures of these components were identified in the PubChem database for predicting component targets using drug mapper. Subsequently, relevant NASH targets were determined through searches in the NCBI gene and gene card databases. The active components were then compared with disease targets to identify common component-disease targets. The common component-disease targets were inputted into the string to construct a protein-protein interaction (PPI) network, which was then visualized using Cytoscape3.8.0 software. To identify the key target of curcumin in the prevention and treatment of nonalcoholic steatohepatitis, a network model was established by Cytoscape3.8.0, incorporating the common targets of component diseases and relevant PPI network data, specifically targeting nonalcoholic steatohepatitis with curcumin’s active ingredient.

The PPI network index was analyzed using the David database, and the enrichments of GO and KEGG pathways related to curcumin in the prevention and treatment of NASH were examined. This study aims to elucidate the subcellular localization, molecular functions, and biological processes of curcumin in the prevention and treatment of NASH, and to investigate the molecular mechanisms underlying its effects on NASH prevention and treatment at the signaling pathway level.

#### Molecular docking

The sub-ligands of curcumin (Cur) and nicotinate-curcumin (NC) were subjected to molecular docking with the AKR1B10 domain using Gold3.0 software. The AKR1B10 protein data (PDBID:4gq0) was utilized for this analysis. Amino acid residues within the docking pocket of AKR1B10 that interact with the ligands include TRP21, VAL48, TYR49, TRP80, HIS111, PHE116, LEU122, PHE123, PRO124, LYS125, GLY129, ASN130, ALA131, ASN161, TRP220, VAL301 and LEU302. Docking molecules include Cur and NC.

#### Immunofluorescence colocalization analysis

HepG2 cells were fixed with 4% paraformaldehyde for 15 min, followed by permeabilization with 0.1% Triton/PBS for 10–20 min at 4℃, based on the subcellular localization of the target protein. Subsequently, the cells were incubated with 35% bovine serum albumin for 1 h, followed by overnight incubation at 4℃. The following day, the cells were washed three times with PBS for 5 min each, and then incubated with the appropriate fluorescent secondary antibody for 1 h. DAPI was used to stain the nuclei after three additional washes with PBS for 5 min each.

#### Data analysis

The data were presented as the mean ± standard deviation (SD) from a minimum of three separate experiments at least. The comparison between two groups was conducted using an independent sample t-test, while single factor analysis of variance (ANOVA) and Dunnett test were employed for intergroup comparisons. A significance level of *P* < 0.05 was deemed statistically significant. All statistical analyses were performed using GraphPad Prism 8.0 software (GraphPad Software, Inc., La Jolla, CA, USA).

## Results

### Effects of nicotinate-curcumin (NC) on hepatic index and pathological alterations in high-fat and high-fructose diet-induced NASH rats

The pharmacodynamic effect of NC was initially assessed in this study (Fig. 1A). Morphological analysis revealed that the liver of the high-fat-high-fructose diet (HFD + Fru) group exhibited fat deposition and a concurrent increase in liver weight compared to the normal group (Ctrl). Treatment with NC, Met, and Cur resulted in an improvement in liver appearance (Fig. 1B), with no significant impact on body weight observed (Fig. 1C). Histological analysis and NAFLD activity score (NAS) revealed that the HFD + Fru group exhibited a higher quantity of vacuolar adipocytes in hepatocytes compared to the control group, suggesting an elevation in lipid droplet accumulation. Treatment with NC mitigated hepatic lobular injury and hepatocyte steatosis. The efficacy of NC was comparable to that of Met and marginally superior to that of Cur (Fig. 1D-E).

### Effects of NC on mitigating hepatic injury and decreasing blood lipid levels in NASH rats induced by HFD + Fru

Serum liver enzymes, including ALT, AST and ALP, are widely recognized as valuable indicators for assessing liver damage. In this study, we found that NC significantly decreased levels of ALT, AST, and ALP in the model group, demonstrating a dose-response relationship. High-dose NC exhibited a therapeutic effect comparable to Met, and slightly superior to Cur（Tab. 1）. Additionally, the NC group showed significantly lower serum levels of TC, TG, LDL-C, and FFA compared to the HFD + Fru group. Notably, NC outperformed Cur and Met in reducing TG and FFA levels. In addition, high-dose NC also improved the level of HDL-C to some extent（Tab. 2）.

**Table 1:** Effects of nicotinate-curcumin (NC) on the levels of ALT, AST and ALP in serum of rats induced by high-fat and high fructose

	Ctrl	HFD + FrU	Met	Cur	NC-L	NC-H
ALT	37.80 ± 2.86	75.10 ± 6.33##	48.00 ± 4.40**	52.10 ± 4.20**	53.00 ± 5.81**	49.00 ± 5.06**
AST	120.00 ± 8.56	200.5 ± 8.80##	160.1 ± 10.47**	165 ± 10.51**	166.9 ± 9.48**	162.7 ± 8.88**
ALP	100.6 ± 6.08	150.4 ± 9.81##	109.8 ± 5.09**	118 ± 10.81**	121.4 ± 12.05**	115.7 ± 9.89**

**Table 2:** Effects of nicotinate-curcumin (NC) on the levels of TC, TG, HDL-C, LDL-C and FFA in serum of rats induced by high-fat and high fructose

	Ctrl	HFD + FrU	Met	cur	NC-L	NC-H
TC	2.46 ± 0.24	7.39 ± 1.22##	4.56 ± 0.45**	5.06 ± 0 48**	5.43 ± 0.53**	4.81 ± 0.33**
TG	1.80 ± 0.21	5.77 ± 0.40##	4.05 ± 0.28**	3.81 ± 8.48**	3.61 ± 0.25**	2.81 ± 0.32**
HDL	1.16 ± 0.20	1.04 ± 0.15	1.26 ± 0.21*	0.91 ± 9.48	1.17 ± 0.20	1.50 ± 0.26**
LDL	0.37 ± 0.05	0.98 ± 0.11##	0.68 ± 0.04**	0.71 ± 7.48**	0.72 ± 0.05**	0.65 ± 0.05**
FFA	0.35 ± 0.02	0.98 ± 0.04##	0.57 ± 0.04**	0.50 ± 5.48**	0.48 ± 0.03**	0.42 ± 0.04**

### The association between AKR1B10/ACCα pathway and NASH, and the potential regulatory effects of NC on NASH

**Fig. 2:**
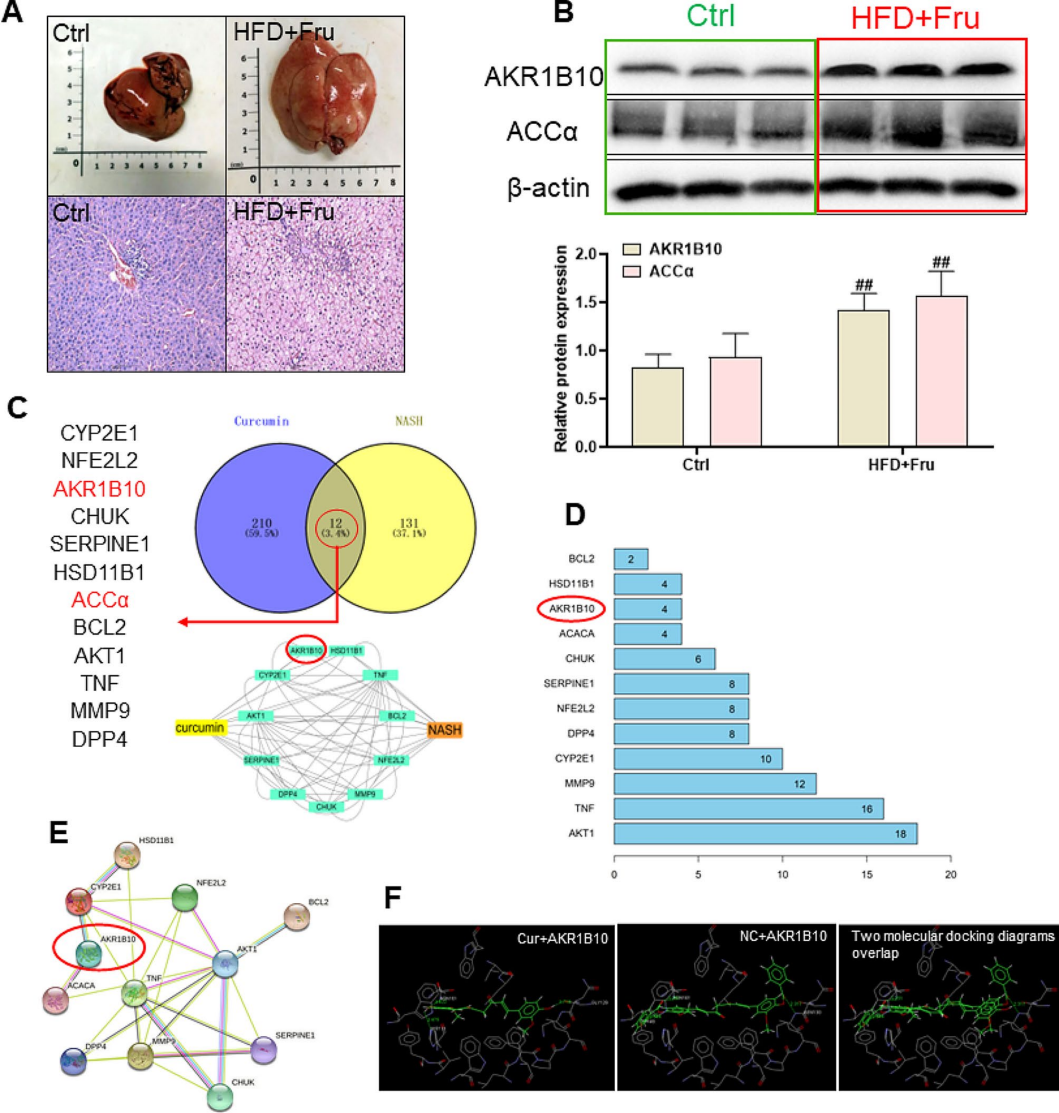
The association between AKR1B10/ACCα pathway and NASH, and the potential regulatory effects of NC on NASH. The hepatic effects of a high-fat and high-fructose-induced NASH rats are depicted as follows: (**A**, top) Contrast images of rat livers between the normal (Ctrl) and high-fat and high-fructose-induced (HFD + Fru) groups; (**A**, lower) Representative images of HE staining in the livers of Ctrl and HFD + Fru groups; (**B**, top) Western blot analysis of AKR1B10 and ACCα proteins; (**B**, lower) Analysis of Western blot band grayscale values. The active constituents of curcumin target NASH-related factors. Twelve genes, including AKR1B10 and ACCα, were involved. (**C**) Network illustrating the active constituents of curcumin and NASH-related targets; (**D**) Interaction network of curcumin’s active components; (**E**) Key genes within the interaction network connecting curcumin’s active constituents and NASH-related targets. Molecular ligands of Cur, NC, and AKR1B10 were subjected to docking using Gold 3.0; (**F**) Presentation of the docking results. The data is expressed in mean ± SD. #*P* < 0.05 and ##*P* < 0.01 vs., control (Ctrl). *P < 0.05 and **P < 0.01 vs. High fat diet + Fructose (HFD + Fru)

Morphological analysis revealed that the liver tissue structure in the control group exhibited normal characteristics, including neatly arranged hepatic lobules and an absence of fat droplets. In contrast, the model group, which was fed a high-fat and high-fructose diet, displayed liver hypertrophy and a yellowish-white appearance, along with fat accumulation as depicted in Fig. 2A (top). Histological examination further indicated a disordered arrangement of hepatic lobules and the presence of hepatocyte vacuoles, indicative of steatosis as shown in Fig. 2A (bottom). The results of Western blotting analysis revealed a significant increase in the expression levels of AKR1B10 and ACCα proteins in the model group, as depicted in Fig. 2B. In order to determine whether NC also has a regulatory effect on AKR1B10/ACCα pathway, we analyzed the interaction of curcumin (the precursor of NC) with NASH, AKR1B10 and ACCα at first by network pharmacology. This involved the examination of Cur targets in TCMSP and TCMID databases, as well as the identification of therapeutic targets relevant to NASH treatment in the NCBI gene bank and gene card database. Through a comparative analysis of Cur and NASH therapy targets, a total of 12 shared targets were identified, with AKR1B10 and ACCα emerging as common targets in the curcumin-NASH relationship. Our analysis of pharmacological data indicates that AKR1B10 is a significant target for Cur in the treatment of NASH, as illustrated in Fig. 2C-E. Molecular docking results suggest that Cur has the ability to interact with histidine residue (HIS111) and glycine residue (GLY129). To further investigate the potential effects of NC on AKR1B10, computer simulation technology was utilized to demonstrate that NC can also form hydrogen bonds with aspartic acid residues (ASN130, ASN161) and tyrosine residues (TYR49) within the AKR1B10 binding pocket. It is worth noting that the binding energy of NC to AKR1B10 is slightly higher than that of Cur to AKR1B10 (Fig. 2F).

### Effects of NC on expression of ABR1B10/ACCα, Malonyl-CoA activity, and synthesis of FFA and TG in NASH rats

**Fig. 3:**
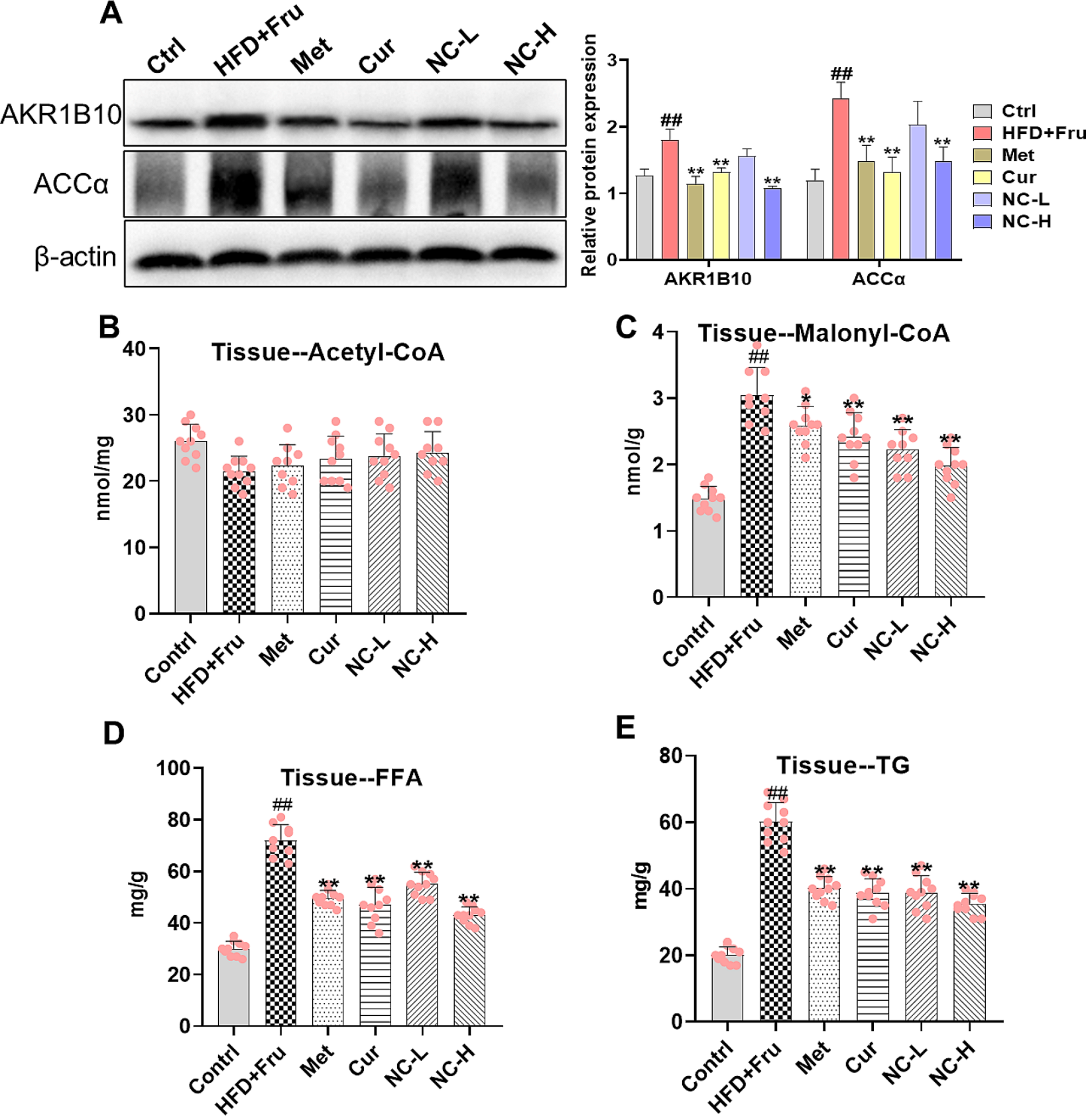
Effects of NC on expression of ABR1B10/ACCα, Malonyl-CoA activity, and synthesis of FFA and TG in NASH rats. (**A**, left) AKR1B10 and ACCα protein bands; (**A**, right) The protein band gray value statistics; (**B**) Acetyl-CoA activity in liver tissue; (**C**) Malonyl-CoA activity in liver tissue; (**D**) Free fatty acid (FFA) content in liver tissue; (**E**) Triglyceride (TG) content in liver tissue. The data is expressed in mean ± SD. ^#^*P* < 0.05 and ^##^*P* < 0.01 vs. control (Ctrl). ^*^P < 0.05 and ^**^P < 0.01 vs. High fat diet + Fructose (HFD + Fru)

In order to elucidate the impact of nicotinate-curcumin (NC) on triglyceride (TG) synthesis by regulating AKR1B10/ACCα pathway, the expression of AKR1B10 and its downstream target protein ACCα was assessed in liver tissue. The results indicated that NC exhibited a significant inhibitory effect on the expression of both AKR1B10 and ACCα with surpassing effects of Cur and Met (Fig. 3A). Malonyl-CoA, a crucial intermediate in TG synthesis derived from Acetyl-CoA, is controlled by ACCα. To elucidate the role of NC in modulating liver lipid metabolism, we assessed the concentrations of Malonyl-CoA, Acetyl-CoA, and their respective metabolites, FFA and TG, in liver tissue. Our findings revealed that acetyl-coenzyme A levels remained unchanged (Fig. 3B). But, the levels of Malonyl-CoA, FFA, and TG were significantly reduced in the NC group, indicating a dose-dependent relationship and superior efficacy compared to Cur and Met treatments (Fig. 3C-E).

### Effects of NC on Malonyl-CoA, total cholesterol, LDL-C and HDL-C in HepG2 cells induced by high fat and glucose

**Fig. 4:**
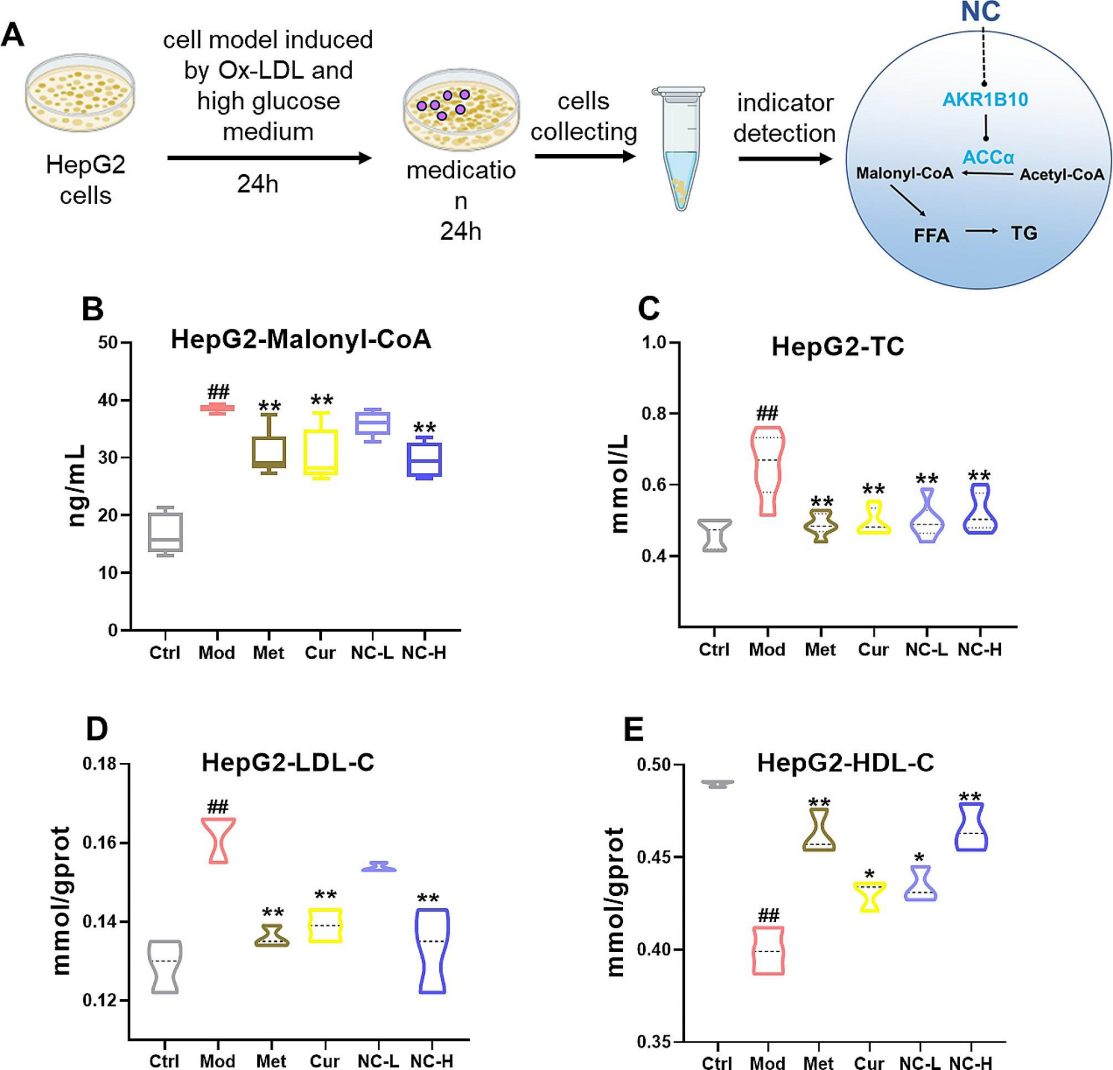
Effects of NC on Malonyl-CoA, total cholesterol, LDL-C and HDL-C in HepG2 cells induced by high fat and glucose. HepG2 cells in normal control group (Ctrl) were cultured in minimum essential medium (MEM). To establish a cellular high-fat model (Mod), HepG2 cells were induced by Ox-LDL (25 µg·ml^− 1^) and cultured in high-glucose medium (DMEM) ; Met:150umol, Cur:40umol, NC-L:20umol, NC-H:40umol; (**A**) Analysis chart of the cell experimental process ; (**B**) Malonyl-CoA activity ; (**C**) Total cholesterol (TC) level in HepG2 cells; (**D**) Low-density lipoprotein cholesterol (LDL-C) content in HepG2 cells; (**E**) High-density lipoprotein cholesterol (HDL-C) content in HepG2 cells. The data is expressed in mean ± SD. ^#^*P* < 0.05 and ^##^*P* < 0.01 vs., control (Ctrl). ^*^P < 0.05 and ^**^P < 0.01 vs. High fat diet + Fructose (HFD + Fru)

HepG2 cells were stimulated with Ox-LDL (25 µg·ml^− 1^), and the MEM medium was substituted with DMEM medium to establish a cellular injury model characterized by high levels of fat and glucose (Fig. 4A). HepG2 cells from Ctrl were cultured in a basic medium with low glucose (MEM). The findings indicated that NC effectively decreased the levels of Malonyl-CoA, TC, and LDL-C in HepG2 cells induced by high fat and glucose(Fig. 4B-E). Specifically, the high-dose NC exhibited a similar effect to the Met group in reducing Malonyl-CoA and LDL-C levels, and slightly outperformed Cur. Simultaneously, NC also demonstrates the capacity to elevate HDL-C levels, with its efficacy being on par with Met and notably superior to Cur, as evidenced by the results of blood analysis in animal trials (Table 2). It is noteworthy that both Cur and NC exhibit minimal influence on Acetyl-CoA levels (Fig. 3B), potentially attributable to the carboxylation of Acetyl-CoA by ACCα and ACCβ, facilitating its involvement in various biochemical pathways, including the tricarboxylic acid cycle. In the process of converting Acetyl-CoA to Malonyl-CoA for incorporation into the synthesis of FFA/TG, only a fraction of Acetyl-CoA undergoes this transformation, resulting in a modest alteration in the overall pool of Acetyl-CoA.

### Effect of NC on the expression of AKR1B10 and ACCα in HepG2 cells induced by high fat and glucose

**Fig. 5:**
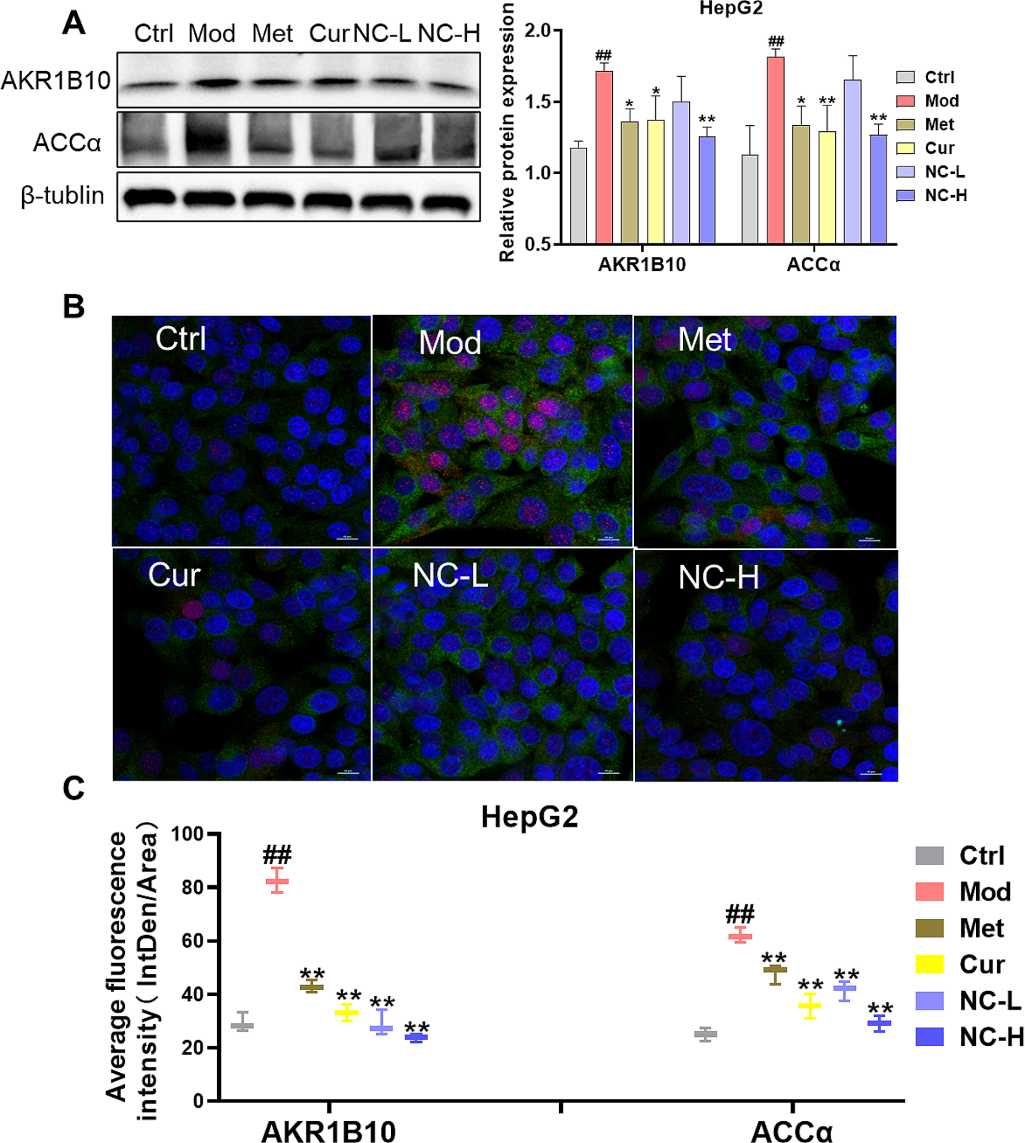
Effect of NC on the expression of AKR1B10 and ACCα in HepG2 cells induced by high fat and glucose. (**A**) Expression of protein (WB) and Analysis of band gray values; (**B**) Co-localization of AKR0B10 and ACCα proteins in HepG2 cells; (**C**) Quantification of average protein fluorescence intensity. The data is expressed in mean ± SD. ^#^*P* < 0.05 and ^##^*P* < 0.01 vs., control (Ctrl). ^*^P < 0.05 and ^**^P < 0.01 vs. High fat diet + Fructose (HFD + Fru)

To further demonstrate the involvement of AKR1B10 and ACCα in the therapeutic potential of nicotinate-curcumin (NC) for NASH, an examination was conducted on the impact of NC on the expression of AKR1B10 and ACCα in HepG2 cell model. The results revealed that NC effectively suppressed the elevation of AKR1B10 and ACCα levels in HepG2 cells induced by high fat and glucose in a manner dependent on dosage. Notably, the efficacy of NC at a high dosage surpassed that of Cur and Met(Fig. 5A-C). These results were further supported by immunofluorescence analysis, which corroborated the western blot findings.

### Effects of NC on ACCα enzyme activity, FFA and TG accumulation in HepG2 cells induced by high fat and glucose, and observe the effects of NC and AKR1B10 inhibitor epalrestat

**Fig. 6:**
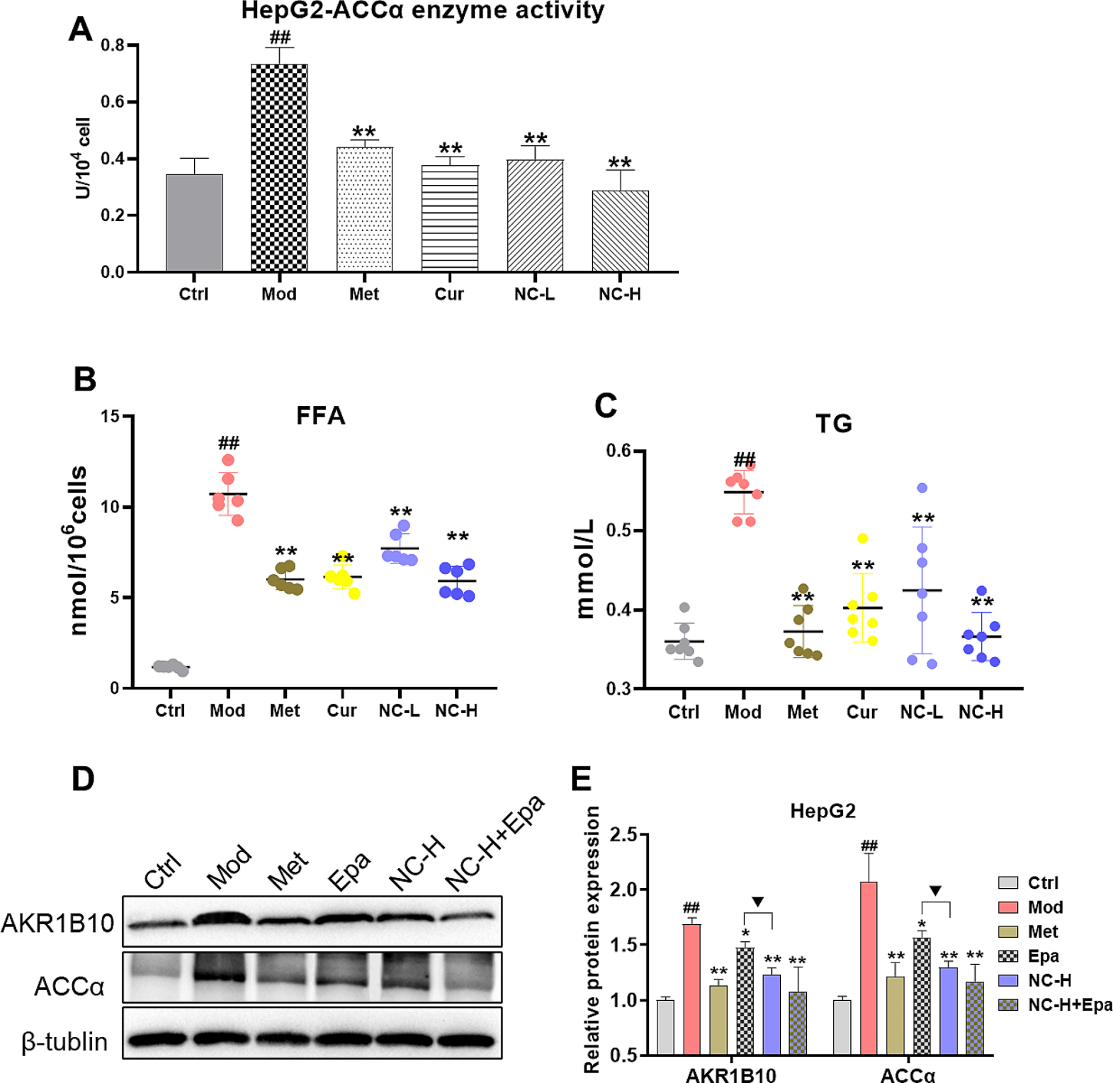
Effects of NC on ACCα enzyme activity, FFA and TG accumulation in HepG2 cells induced by high fat and glucose, and observe the effects of NC and AKR1B10 inhibitor epalrestat. (**A**) The activity of ACCα enzyme; (**B**) The concentration of free fatty acid (FFA) in HepG2 cells; (**C**) The concentration of triglyceride (TG) in HepG2 cells. (**D**) Expression of protein (WB); (**E**) Analysis of band gray values. The data is expressed in mean ± SD. ^#^*P* < 0.05 and ^##^*P* < 0.01 vs., control (Ctrl). ^*^P < 0.05 and ^**^P < 0.01 vs. High fat diet + Fructose (HFD + Fru). ^▼^*P* < 0.05 and ^▼▼^*P* < 0.01 vs. epalrestat (Epa)

To further substantiate the role of NC in the regulation of FFA and TG synthesis via the AKR1B10/ACCα pathway, we conducted a direct assessment of ACCα enzyme activity, compare NC with the AKR1B10 inhibitor epalrestat (Epa), and set up another combination of NC and Epa. Our results demonstrate that the NC significantly exhibited ACCα activity(Fig. 6A) and subsequent synthesis of FFA and TG with a dose-dependent manner (Fig. 6B). More importantly, NC exhibited a more pronounced inhibitory effect on TG synthesis compared to Cur and Met (Fig. 6C). For the inhibitory effect of Epa, NC has a stronger inhibitory effect on AKR1B10 (Fig. 6D-E), suggesting that there may be other regulatory mechanisms in NC, and further research will continue to explore them.

## Discussion

NASH is a condition that arises from the progression of NAFLD, characterized by the continuous accumulation of TG in hepatocytes leading to fat infiltration and inflammation [33]. This progression can result in the development of NASH, hepatocyte vesicular degeneration, diffuse lobulitis, fibrosis and so on [1]. If left untreated, NASH has the potential to advance to cirrhosis and hepatocellular carcinoma [34]. The evolution from simple steatosis to NASH in NAFLD is marked by the accumulation of fat and tissue inflammation [35]. When fatty acid catabolism is found to be inadequate in addressing lipid overload in the liver, toxic fatty acid derivatives are generated, leading to the activation of the inflammasomes [36]. The heightened presence of endoplasmic reticulum, oxidative stress, and hepatocyte apoptosis play a role in the advancement of steatosis to NASH [37–39]. Furthermore, signals emanating from stressed or injured hepatocytes, in conjunction with activated macrophages, prompt resident hepatic stellate cells to transition into myofibroblasts and generate an overabundance of matrix proteins, thereby exacerbating the severity of NASH [40, 41].

Numerous studies reported that some drugs [42, 43], such as vitamin E, pioglitazone, acetylcholic acid, can utilize in clinical treatment of NASH [44–46]. However, research indicates that prolonged use of vitamin E may be linked to hemorrhagic stroke and heightened prostate cancer risk in otherwise healthy males [47]. Pioglitazone is associated with adverse effects such as weight gain, fluid retention, bone density reduction, and a potential elevated risk of bladder cancer [48, 49]. Ocaliva (OCA), initially sanctioned for primary biliary cholangitis (PBC) treatment, has the potential to become an FDA-approved medication for NASH. However, the safety profile of OCA necessitates further investigation, and currently, there is only approved resmetirom as the world’s first drug for treating NASH, and still has approved drugs for NASH halting the transition from NAFLD to NASH [50, 51]. It is crucial to note that once NASH develops, reversing the condition becomes challenging and often requires a liver transplant. Therefore, early intervention strategies focus on mitigating steatosis through lipid balance regulation to impede NAFLD progression to NASH and severe liver complications.

Curcumin (Cur) demonstrates promise for addressing nonalcoholic fatty liver disease due to its diverse health-enhancing attributes, notably its anti-inflammatory and antioxidant properties [52, 53]. We synthesized nicotinate-curcumin (NC) through the incorporation of a nicotinic acid group into Cur, thereby enhancing its solubility and bioavailability. Prior studies indicate that NC has the potential to lower blood lipid levels and safeguard liver function [25]. Intriguingly, findings suggest that NC surpasses Cur in its ability to modulate lipid metabolism and combat atherosclerosis [24–30]. In this study, we assessed the preventative and therapeutic effects of NC on NASH in rats induced by HFD + Fru and conducted a comparison with Cur and metformin. The results indicated that NC was effective in reducing the levels of ALT, AST, and ALP in HFD + Fru-treated rats, with NC-H (administered at the equivalent dose as Cur) exhibiting superior efficacy compared to Cur (Table 2).

The involvement of FFA and TG is significant in the pathogenesis of NASH [54, 55]. The progressive accumulation of FFA within the liver hastens the progression from simple steatosis to steatohepatitis [56]. Patients with NASH exhibit heightened levels of FFA within the liver and in circulation [57]. Elevated FFA levels, in conjunction with de novo lipogenesis (DNL) in the bloodstream, facilitate lipid accumulation and stimulate TG synthesis in the liver, ultimately contributing to the development of insulin resistance [58]. In this experiment, we observed that NC effectively improved dyslipidemia in NASH rats, particularly reduced serum levels of FFA and TG. The therapeutic impact of NC was superior to that of Cur and Met, as indicated in Table 1.

Several studies have demonstrated that Acetyl-CoA carboxylase (ACCα) plays a crucial role in the regulation of de novo fatty acid synthesis, with ACC inhibitors showing potential in alleviating non-alcoholic fatty liver disease and liver insulin resistance [59, 60]. Additionally, AKR1B10 has been identified as a differentially expressed gene in human NASH [9]. In a physiological state, AKR1B10 is primarily expressed in gastric and intestinal epithelial cells, with minimal to no expression in other tissues; however, in NASH, AKR1B10 expression is aberrantly upregulated [61, 62]. AKR1B10 is involved in the regulation of ACCα, a key enzymatic step in fat formation. ACCα catalyzes the condensation of adenosine triphosphate-dependent Acetyl-CoA with carbonate to form Malonyl-CoA, a crucial molecule in the synthesis and metabolism of fatty acids. Studies have shown that phosphorylation and subsequent inactivation of ACCα can enhance fatty acid oxidation, decrease Malonyl-CoA levels, and protect mice from liver steatosis induced by a high fructose diet. Additionally, in a rat NASH model, up-regulation of AKR1B10 expression was observed, while treatment with the AKR1B10 inhibitor sulindac significantly reduced its expression [9, 12, 13]. In this experiment, the therapeutic effect of NC on NASH rats was observed, revealing its ability to effectively improve the abnormal increase of ALT, ALP, AST, TC, and LDL induced by high-fat and high-fructose. Additionally, western blot and immunofluorescence results demonstrated a significant increase in protein levels of AKR1B10 and ACCα in NASH rats and HepG2 cells, which were subsequently decreased by NC treatment. Furthermore, levels of Malonyl-CoA, TG, and FFA decreased in a dose-dependent manner following NC treatment. (Fig. 4). The aforementioned findings validate our scientific hypothesis regarding the potential therapeutic effects of NC in alleviating NASH. Specifically, NC has been shown to decrease the carboxylation of Acetyl-CoA to Malonyl-CoA by downregulating the expression of AKR1B10 and ACCα proteins, subsequently reducing the synthesis of FFA and TG. Additionally, utilizing network pharmacology and molecular docking techniques, AKR1B10 and ACCα have been identified as potential therapeutic targets for Cur in the treatment of NASH (Fig. 3C). Both Cur and NC exhibit potential for binding to AKR1B10 protein residues, but with the binding energy of NC to AKR1B10 slightly surpassing that of Cur (Fig. 3F). Whereafter, we further demonstrated that the inhibitory efficacy of NC on AKR1B1 was superior to that of Cur at equivalent concentrations.

The incidence of NASH is positively correlated with the rise in rates of obesity and type 2 diabetes [63]. NASH is caused by liver fat accumulation due to insulin resistance, visceral obesity, and/or metabolic syndrome traits, and long-term accumulation can lead to liver fibrosis [64, 65]. The development of liver fibrosis can progress to cirrhosis and create a conducive environment for the development of hepatocellular carcinoma. Individuals with obesity and diabetes are identified as high-risk populations for NASH [66]. Due to the widespread occurrence and severe consequences of NASH, there is a concerted effort to develop efficacious treatment modalities. Prior research has demonstrated the in vivo stability of NC. Through network pharmacology screening and molecular docking, this study posits that NC may interact with AKR1B10. Furthermore, animal and cellular experiments have shown that NC can modulate ACCα enzyme activity by downregulating AKR1B10 protein expression, leading to decreased Acetyl-CoA carboxylation and subsequent reduction in free fatty acid and lipid synthesis. Following the addition of the AKR1B10 inhibitor, similar inhibitory effects were observed, with NC demonstrating a superior inhibitory effect, suggesting that NC may potentially act through alternative pathways. Examining the heightened biomarkers present in patients with non-alcoholic steatohepatitis (NASH) serves as a method for identifying potential therapeutic targets and medications, including Transcriptional Activator Like 1 (TAZ, also known as WWTR1) [67], plasma bile acids [68], AKR1B10 [9], and vascular regulatory factor Run Related Transcription Factor 1 (RUNX1) [69], all of which are elevated in NASH. Our study specifically focuses on the alterations in AKR1B10 expression, which impacts triglyceride synthesis, with the aim of halting the progression of non-alcoholic fatty liver disease (NAFLD) to NASH and offering insights for the advancement of clinical drug development.

## Conclusion

**Fig. 7:**
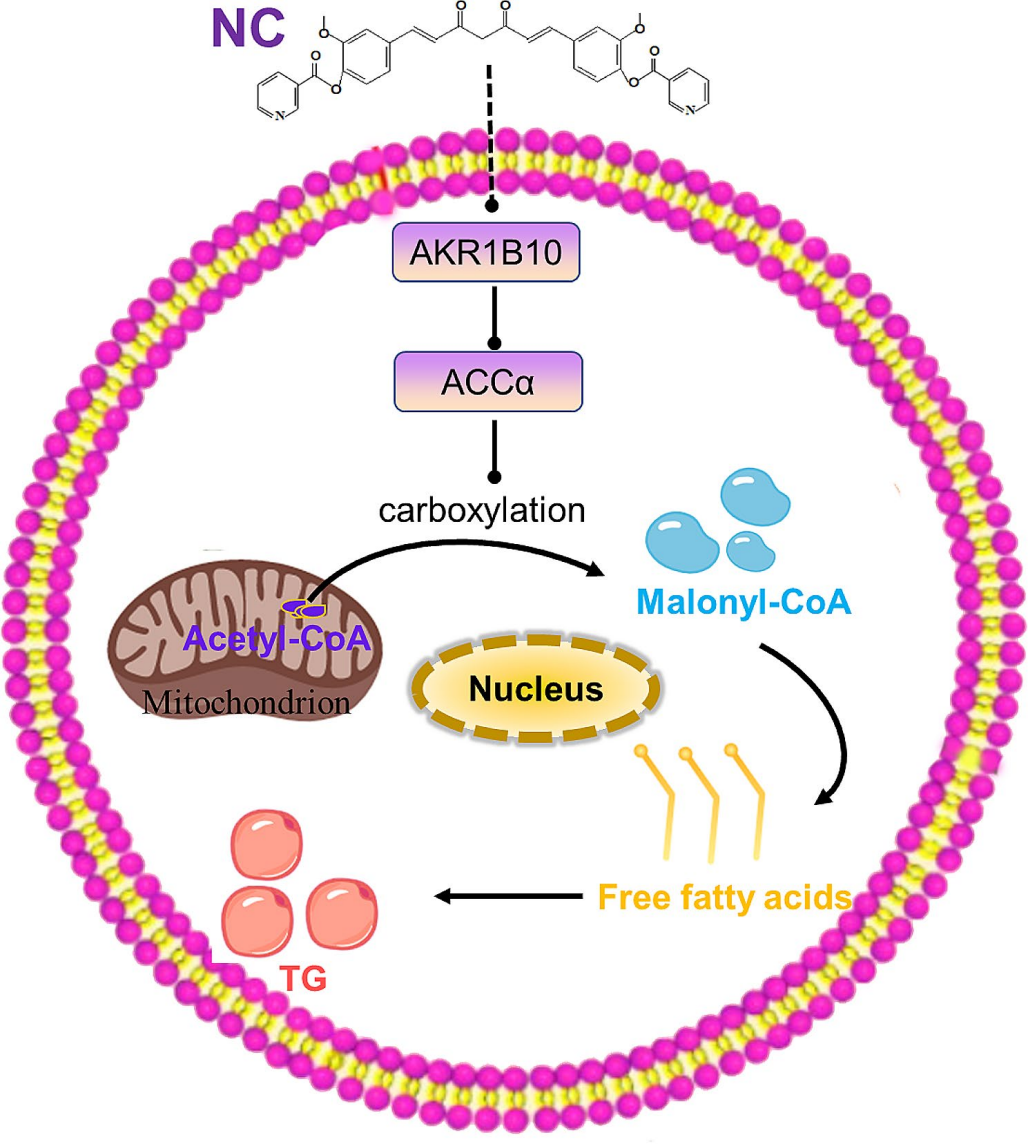
The mechanism diagram of NC via AKR1B10/ACCα pathway to reduce FFA and TG synthesis. Nicotinic-curcumin (NC) inhibits the expression of AKR1B10 in liver tissue, leading to the downregulation of ACCα levels. This regulation further slows down the carboxylation of acetyl-CoA, resulting in a reduction in the generation of succinyl-CoA. Ultimately, the action of NC inhibits the synthesis of free fatty acids (FFA) and triglycerides (TG), significantly preventing and treating non-alcoholic steatohepatitis (NASH)

The accumulation of FFA and TG in liver cells play a key role in the progression of NAFLD to NASH, which is regulated by the AKR1B10/ACCα/Acetyl-CoA/Malonyl-CoA axis. NC effectively inhibits the synthesis and accumulation of FFA and TG, and then improves liver function and lipid metabolism disorders, ultimately treats NASH. The mechanism of NC may be attributed to its regulation of the AKR1B10/ACCα/Acetyl-CoA/Malonyl-CoA axis (Fig. 7). This research serves as a basis for future investigations into the pharmacological properties and clinical utility of nicotinate-curcumin.

### Electronic supplementary material

Below is the link to the electronic supplementary material.

Supplementary Material 1

## Data Availability

No datasets were generated or analysed during the current study.

## Abbreviations

NASH: Nonalcoholic steatohepatitis
NAFLD: Nonalcoholic fatty liver disorder
AKR1B10: Aldo-Keto reductase family 1 member B10
ACCα: Acetyl-CoA carboxylase
NC: Nicotinate-curcumin
Cur: Curcumin
Met: Metformin
HFD + Fru: The high-fat-high-fructose diet
FFA: Free fatty acid
PPI: Protein–protein Interaction
ALT: Alanine aminotransferase
AST: Aspartate aminotransferase
ALP: Alkaline phosphatase
TC: Total cholesterol
TG: Triglyceride
LDL-C: Low-density lipoprotein cholesterol
HDL-C: High-density lipoprotein cholesterol
ELISA: Enzyme-linked immunosorbent assay
OCA: Ocaliva
