# Supplementary material for: Nicotinate-curcumin improves NASH by inhibiting the AKR1B10/ACCα-mediated triglyceride synthesis
Source: Lipids Health Dis. 2024 Jun 27;23:201. doi: 10.1186/s12944-024-02162-5 (PMC11210137; doi:10.1186/s12944-024-02162-5)
Supplement: Supplementary file 1 — Supplementary Material 1 [file 12944_2024_2162_MOESM1_ESM.pdf]

SPRINGER NATURE

Change of authorship request form - Journals (pre-acceptance)

Important information. Please read.

- This form should be used by authors to request any change in authorship (adding/deleting authors) including changes in corresponding authors. This form should not be used for name changes. Please fully complete all sections. Use black ink and block capitals and provide each author's full name with the given name first followed by the family name.

- By signing this declaration, all authors guarantee that the order of the authors are in accordance with their scientific contribution, if applicable as different conventions apply per discipline, and that only authors have been added who made a meaningful contribution to the work.

- Please note, in author collaborations where there is formal agreement for representing the collaboration, it is sufficient for the representative or legal guarantor (usually the corresponding author) to complete and sign the Authorship Change Form on behalf of all authors, **next to the added/removed author(s). (Complete Section 3, followed by Section 6.)**
  In author collaborations where there is no formal agreement for representing the collaboration and **there are more than 10 authors**, one may sign for all, provided the signer appends correspondence that attests that each of the authors have agreed to the change **and the added/removed authors sign the form. (Complete Section 3, followed by Section 6.)**

- Please note, we cannot investigate or mediate any authorship disputes. If you are unable to obtain agreement from all authors (including those who you wish to be removed) you must refer the matter to your institution(s) for investigation. Please inform us if you need to do this.

- If you are not able to return a fully completed form within **30 days** of the date that it was sent to the author requesting the change, we may have to withdraw your manuscript. We cannot publish manuscripts where authorship has not been agreed by all authors (including those who have been removed).

- Incomplete forms will be rejected.

- Please return/upload this form, fully completed, to the Journals Editorial Office. The Journal and/or Publisher will consider the information you have provided to decide whether to approve the proposed change in authorship. We may decide to contact your institution for more information or undertake a further investigation, if appropriate, before making a final decision.

SPRINGER NATURE Springer Nature is one of the world's leading global research, educational and professional publishers, created in May 2015 through the combination of Nature Publishing Group, Palgrave Macmillan, Macmillan Education and Springer Science+Business Media.

SPRINGER NATURE

Change of authorship request form - Journals (pre-acceptance)

Section 1: Please provide the current title of manuscript

Manuscript ID no.: 691261f9-c3c4-48c1-9d8c-f2d19268b586

Title: Nicotinate-curcumin improves NASH by inhibiting the AKR1B10/ACCα-mediated triglyceride synthesis

Section 2: Please provide the previous authorship, in the order shown on the manuscript before the changes were introduced. Please indicate the corresponding author by adding (CA) behind the name.

	First name(s)	Family name	ORCID or SCOPUS id, if available
1st author	Xiu-lian	Lin	
2nd author	Ya-ling	Zeng	
3rd author	Jie	Ning	
4th author	Zhe	Cao	
5th author	Lan-lan	Bu	
6th author	Wen-jing	Liao	
7th author	Zhi-min	Zhang	
8th author	Tan-jun	Zhao	
9th author	Rong-geng	Fu	
10th author	Xue-feng	Yang	
11th author	Jian-guo	Zeng	
12th author	De-liang	Cao	
13th author	Cai-ping	Zhang	
14th author	Duan-fang	Liao	
15th author	Yong-zhen	Gong	
16th author	Ya-mei	Li	

Please use an additional sheet if there are more than 10 authors.

SPRINGER NATURE Springer Nature is one of the world's leading global research, educational and professional publishers, created in May 2015 through the combination of Nature Publishing Group, Palgrave Macmillan, Macmillan Education and Springer Science+Business Media.

SPRINGER NATURE

Change of authorship request form - Journals (pre-acceptance)

Section 3: Please provide a justification for change. Please use this section to explain your reasons for changing the authorship of your manuscript, e.g. what necessitated the change in authorship? Please refer to the (journal) policy pages for more information about authorship. Please explain why omitted authors were not originally included and/or why authors were removed on the submitted manuscript.

ppear on the manuscript. Please indicate the corresponding author byadding (CA) behind the name. <u>If the Corresponding Author has changed, please indicate the reason under section 3</u>.

We want to change the author order of the paper,

Jian-guo Zeng was the initiator of the entire study and participated in the design and revision of the article. Therefore, Jian-guo Zeng was listed as one of the corresponding authors and was listed as the first corresponding author;

Yong-zhen Gong was changed to one of the authors because she read the entire text and corrected the manuscript;

Li-mei Lin was added as one of the authors because she contributed to the writing and review of the revised manuscript.

We have communicated with all authors regarding the change in author order and obtained their consent.

	First name(s)	Family name (this name will appear in full on the final publication and will be searchable in various abstract and indexing databases)	Affiliated institute	E-mail address
1st author	Xiu-lian	Lin	Key Laboratory for Quality Evaluation of Bulk Herbs of Hunan Province, Hunan University of Chinese Medicine, Changsha, Hunan, 410208, China	1742825988@qq.com
2nd author	Ya-ling	Zeng	Key Laboratory for Quality Evaluation of Bulk Herbs of Hunan Province, Hunan University of Chinese Medicine, Changsha, Hunan, 410208, China	597557388@qq.com
3rd author	Jie	Ning	Key Laboratory for Quality Evaluation of Bulk Herbs of Hunan Province, Hunan University of Chinese Medicine, Changsha, Hunan, 410208, China	2904660807@qq.com
4th author	Zhe	Cao	Hunan Laituofu Biotechnology Co., Ltd, Jinzhou New District, Ningxiang, 410604, Hunan, China	731397600@qq.com

5th author	Lan-lan	Bu	Key Laboratory for Quality Evaluation of Bulk Herbs of Hunan Province, Hunan University of Chinese Medicine, Changsha, Hunan, 410208, China	1240607651@qq.com
6th author	Wen-jing	Liao	Key Laboratory for Quality Evaluation of Bulk Herbs of Hunan Province, Hunan University of Chinese Medicine, Changsha, Hunan, 410208, China	Foxyusa@163.com
7th author	Zhi-min	Zhang	Key Laboratory for Quality Evaluation of Bulk Herbs of Hunan Province, Hunan University of Chinese Medicine, Changsha, Hunan, 410208, China	zzm@hnucm.edu.cn
8th author	Tan-jun	Zhao	Key Laboratory for Quality Evaluation of Bulk Herbs of Hunan Province, Hunan University of Chinese Medicine, Changsha, Hunan, 410208, China	350806259@qq.com
9th author	Rong-geng	Fu	Key Laboratory for Quality Evaluation of Bulk Herbs of Hunan Province, Hunan University of Chinese Medicine, Changsha, Hunan, 410208, China	22158421@qq.com
10th author	Xue-feng	Yang	Hunan Provincial Clinical Research Center for Metabolic Associated Fatty Liver Disease, Hengyang, Hunan, 421002, China	yxf9988@126.com
11th author	Yong-zhen	Gong	Key Laboratory for Quality Evaluation of Bulk Herbs of Hunan Province, Hunan University of Chinese Medicine, Changsha, Hunan, 410208, China	yzgong@hnucm.edu.cn
12th author	Li-mei	Lin	Key Laboratory for Quality Evaluation of Bulk Herbs of Hunan Province, Hunan University of Chinese Medicine, Changsha, Hunan, 410208, China	limei_lin@hnucm.edu.cn
13th author	De-liang	Cao	Key Laboratory for Quality Evaluation of Bulk Herbs of Hunan Province, Hunan University of Chinese Medicine, Changsha, Hunan, 410208, China<br>Hunan Laituofu Biotechnology Co., Ltd, Jinzhou	1814589590@qq.com

---
SPRINGER NATURE Springer Nature is one of the world's leading global research, educational and professional publishers, created in May 2015 through the combination of Nature Publishing Group, Palgrave Macmillan, Macmillan Education and Springer Science+Business Media.

			New District, Ningxiang, 410604, Hunan, China	
14th author	Cai-ping	Zhang	Department of Biochemistry & Molecular Biology, Hengyang Medical School, University of South China, Hengyang, 421001, Hunan, China	zhangcpin@163.com
15th author	Duan-fang	Liao	Key Laboratory for Quality Evaluation of Bulk Herbs of Hunan Province, Hunan University of Chinese Medicine, Changsha, Hunan, 410208, China<br>Hunan Provincial Clinical Research Center for Metabolic Associated Fatty Liver Disease, Hengyang, Hunan, 421002, China	dfliao@hnucm.edu.cn
16th author	Ya-mei	Li	Key Laboratory for Quality Evaluation of Bulk Herbs of Hunan Province, Hunan University of Chinese Medicine, Changsha, Hunan, 410208, China	yameili@hnucm.edu.cn
17th author	Jian-guo	Zeng (corresponding author)	Key Laboratory for Quality Evaluation of Bulk Herbs of Hunan Province, Hunan University of Chinese Medicine, Changsha, Hunan, 410208, China<br>Hunan Key Laboratory of Traditional Chinese Veterinary Medicine, Hunan Agricultural University, Changsha, 410128, Hunan, China	zengjianguo@hunau.edu.cn

Please use an additional sheet if there are more than 10 authors.

SPRINGER NATURE Springer Nature is one of the world's leading global research, educational and professional publishers, created in May 2015 through the combination of Nature Publishing Group, Palgrave Macmillan, Macmillan Education and Springer Science+Business Media.

Section 5: Author contribution, Acknowledgement and Disclosures. Please use this section to provide a new disclosure statement and, if appropriate, acknowledge any contributors who have been removed as authors and ensure you state what contribution any new authors made (if applicable per the journal or book (series) policy). Please ensure these are updated in your manuscript - after approval of the change(s) - as our production department will not transfer the information in this form to your manuscript.

New acknowledgements:

We would like to express our gratitude to all those who helped us during the writing of this manuscript. We thank all the peer reviewers for their opinions and suggestions.

New Disclosures (financial and non-financial interests, funding):

This work was supported by Hunan Changzhutan national independent innovation zone special project (No.2018XK2106); Hunan Science and Technology Innovation Team Project (No.2021RC4064) and Key Discipline Project on Chinese Pharmacology of Hunan University of Chinese Medicine (No.202302); Outstanding Youth Program of Hunan Provincial Department of Education (No.21B0397); Natural Science Foundation of Hunan University of Chinese Medicine (NO. Z2023XJYB12)

New Author Contributions statement (if applicable per the journal policy):

JGZ, YML and DFL conceived the idea. XLL, YLZ, JN, ZC and CPZ completed the experiments and wrote the manuscript; LLB, WJL, ZMZ, TJZ, RGF and XFY conducted the data collection and analysis. JGZ, DLC and YZG read through and corrected the manuscript. JGZ, LML made major contributions in guiding manuscript revisions. All authors read and approved the final manuscript.

State 'Not applicable' if there are no new authors.

SPRINGER NATURE Springer Nature is one of the world's leading global research, educational and professional publishers, created in May 2015 through the combination of Nature Publishing Group, Palgrave Macmillan, Macmillan Education and Springer Science+Business Media.

SPRINGER NATURE

Change of authorship request form - Journals (pre-acceptance)

Section 6: Declaration of agreement. *All* authors, unchanged, new and removed *must* sign this declaration.
(NB: Please print the form, (docu)-sign and return/upload a scanned copy. Please note that signatures that have been inserted as an image file are acceptable as long as it is handwritten. Typed names in the signature box are unacceptable.) *** Please delete as appropriate. Delete all of the bold if you were on the original authorship list and are remaining as an author.**

	First name	Family name		Signature	Date
1st author	Xiu-lian	Lin	I agree to the proposed new authorship shown in section 4 **/and the addition/removal*of my name to the authorship list** /and the proposed change in corresponding author	Xiu-lian Lin	13 May 2024
2nd author	Ya-ling	Zeng	I agree to the proposed new authorship shown in section 4 **/and the addition/removal*of my name to the authorship list** /and the proposed change in corresponding author	Yaling Zeng	13 May 2024
3rd author	Jie	Ning	I agree to the proposed new authorship shown in section 4 **/and the addition/removal*of my name to the authorship list** /and the proposed change in corresponding author	Jie Ning	13 May 2024
4th authors	Zhe	Cao	I agree to the proposed new authorship shown in section 4 **/and the addition/removal*of my name to the authorship list** /and the proposed change in corresponding author	Zhe Cao	13 May 2024
5th author	Lan-lan	Bu	I agree to the proposed new authorship shown in section 4 **/and the addition/removal*of my name to the authorship list** /and the proposed change in corresponding author	Lan-lan Bu	13 May 2024
6th author	Wen-jing	Liao	I agree to the proposed new authorship shown in section 4 **/and the addition/removal*of my name to the authorship list** /and the proposed change in corresponding author	Wen-jing Liao	13 May 2024
7th author	Zhi-min	Zhang	I agree to the proposed new authorship shown in section 4 **/and the addition/removal*of my name to the authorship list** /and the proposed change in corresponding author	Zhi-min Zhang	13 May 2024

SPRINGER NATURE Springer Nature is one of the world's leading global research, educational and professional publishers, created in May 2015 through the combination of Nature Publishing Group, Palgrave Macmillan, Macmillan Education and Springer Science+Business Media.

8th author	Tan-jun	Zhao	I agree to the proposed new authorship shown in section 4 **/and the addition/removal*of my name to the authorship list** /and the proposed change in corresponding author	Tan-jun Zhao	13 May 2024
9th author	Rong-geng	Fu	I agree to the proposed new authorship shown in section 4 **/and the addition/removal*of my name to the authorship list** /and the proposed change in corresponding author	Rong-geng Fu	13 May 2024
10th author	Xue-feng	Yang	I agree to the proposed new authorship shown in section 4 **/and the addition/removal*of my name to the authorship list** /and the proposed change in corresponding author	Xue-feng Yang	13 May 2024
11th author	Yong-zhen	Gong	I agree to the proposed new authorship shown in section 4 **/and the addition/removal*of my name to the authorship list** /and the proposed change in corresponding author	Yong-zhen Gong	13 May 2024
12th author	Li-mei	Lin	I agree to the proposed new authorship shown in section 4 **/and the addition/removal*of my name to the authorship list** /and the proposed change in corresponding author	Li-mei Lin	13 May 2024
13th author	De-liang	Cao	I agree to the proposed new authorship shown in section 4 **/and the addition/removal*of my name to the authorship list** /and the proposed change in corresponding author	De-liang Cao	13 May 2024
14th author	Cai-ping	Zhang	I agree to the proposed new authorship shown in section 4 **/and the addition/removal*of my name to the authorship list** /and the proposed change in corresponding author	Cai-ping zhang	13 May 2024
15th author	Duan-fang	Liao	I agree to the proposed new authorship shown in section 4 **/and the addition/removal*of my name to the authorship list** /and the proposed change in corresponding author	Duan-fang Liao	13 may 2024

SPRINGER NATURE Springer Nature is one of the world's leading global research, educational and professional publishers, created in May 2015 through the combination of Nature Publishing Group, Palgrave Macmillan, Macmillan Education and Springer Science+Business Media.

16th author	Ya-mei	Li	I agree to the proposed new authorship shown in section 4 /**and the addition/removal*of my name to the authorship list** /and the proposed change in corresponding author	Ya-mei Li	13 May 2024.
17th author	Jian-guo	Zeng (corresponding author)	I agree to the proposed new authorship shown in section 4 /**and the addition/removal*of my name to the authorship list** /and the proposed change in corresponding author	Jian-guo Zeng	13 May 2024

Please use an additional sheet if there are more than 10 authors.

In case of author collaborations with formal agreement:

	Name of consortium/consortia	First name	Family name		Signature	Date
Representative/legal guarantor				I agree to the proposed new authorship shown in section 4 /**and the addition/removal*of my name to the authorship list** /and the proposed change in corresponding author		

Both added/removed authors should complete the information in the first table under Section 6.

---- End of form ----

SPRINGER NATURE Springer Nature is one of the world's leading global research, educational and professional publishers, created in May 2015 through the combination of Nature Publishing Group, Palgrave Macmillan, Macmillan Education and Springer Science+Business Media.
